# Who needs beds?

**DOI:** 10.1007/s40037-014-0146-8

**Published:** 2014-10-10

**Authors:** Peter Cantillon, Tim Dornan

**Affiliations:** 1 Discipline of General Practice, Clinical Science Institute, National University of Ireland, Galway, Costello Road, Galway, Ireland; 2 School of Health Professions Education, Maastricht University, Maastricht, the Netherlands

To the Editor,

We wish to respond to two recent articles highlighting a decline in the frequency of bedside teaching and advocating a return to this traditional clinical education set piece [1, 2] We agree with the assertion that bedside teaching has, in the past, proved to be an important form of clinical education through which learners gain professional, communication, physical examination and reasoning skills. However, we question the validity of the authors’ claim that a decline in the prevalence of bedside teaching has contributed to a deterioration in the clinical skills of new graduates. The evidence provided for this assertion is largely based on opinion pieces whereas recent surveys of readiness for practice indicate that graduates are increasingly well prepared for clinical practice [3].

We would also like to take issue with the words ‘bedside’ and ‘teaching’. Teaching at patients’ bedsides has a particular cultural and historical resonance for medical educators because it represents what Shulman [4] would call a signature pedagogy of their own apprenticeship learning experience. However, given the rapidly changing nature of hospital clinical environments, it is important to recognize that clinical education, (i.e. education occurring in the presence of patients) can and does occur in a variety of settings without the necessity for the patient to be supine in a bed. Clinical learning now occurs in a range of contexts including investigative settings, ambulatory clinics, therapeutic areas, (e.g. physiotherapy) and in the community [5]. The term ‘bedside’ has become increasingly redundant because that is not where patients spend much of their time, even in hospitals.

‘Teaching’ is a word that, in the eyes of some, positions the teacher as a transmitter of knowledge that is ‘acquired’ by learners. There is an increasing recognition that learning is a much more complex process than the transmission/acquisition metaphor suggests [6]. Increasingly, researchers of clinical education conceptualize becoming a doctor as a process of identity formation and professional development that occurs through participation in practice [5]. Thus, the term ‘learning’ has become more important than ‘teaching’. Clinical learning is shaped fundamentally by the relationship between learners, teachers, and the environments in which they interact. Being an effective teacher or learner is about recognizing and making the most of opportunities (affordances) that present themselves in a variety of settings [7]. Issues such as learner safety, observation of student participation, the judicious and deliberate use of feedback, the identification and follow-up of learning goals are all more important determinants of clinical education quality than whether the teaching occurs at a bedside or in a primary health care centre, where there are no beds [5].

Perhaps what we need is not a retreat to the bedside, but rather a new discourse of clinical education based on maximizing opportunities for learning and the legitimization of learners as genuine participants in clinical practice, wherever that practice may be.
